# P-927. Impact of an Antimicrobial Stewardship Bundle on the Treatment of Urinary Tract Infections in Geriatric Patients

**DOI:** 10.1093/ofid/ofaf695.1131

**Published:** 2026-01-11

**Authors:** Elly R Sherman, L Taylor Corbin, Rachael W Petry, Milner Staub, Maria N Muehrcke, Laura Bobbitt

**Affiliations:** Vanderbilt University Medical Center, Nashville, TN; Belmont University College of Pharmacy and Health Sciences, Nashville, Tennessee; Vanderbilt University Medical Center, Nashville, TN; Vanderbilt University Medical Center, VA Tennessee Valley Healthcare System, Nashville, TN; Vanderbilt University Medical Center, Nashville, TN; Vanderbilt University Medical Center, Nashville, TN

## Abstract

**Background:**

Urinary tract infections (UTIs) are a common indication for hospital admission in geriatric patients but are often misdiagnosed, especially in those with altered mental status. Overtreating UTIs can lead to antimicrobial resistance and adverse drug effects. Many institutions, including Vanderbilt University Hospital, have implemented antimicrobial stewardship initiatives to mitigate these risks. This retrospective, single-center analysis aimed to assess whether antimicrobial prescribing changed after the implementation of a geriatrics-specific antimicrobial stewardship bundle with a treatment algorithm.

**Methods:**

All patients were ≥ 65 years of age, admitted to the geriatrics teaching service, and had a urinalysis ordered during admission. Patients with neurogenic bladder, altered urologic anatomy, concomitant infection, antibiotics immediately prior to admission, or a transition to comfort care within 48 hours of admission were excluded. The primary outcome was days of antimicrobial therapy per 1000 patient days. Secondary outcomes included rates of antibiotic therapy discontinuation in asymptomatic bacteriuria, antibiotic duration, *C. difficile* colitis rates within 90 days, and proportion of patients with inappropriate UTI diagnoses.

**Results:**

Of 372 patient encounters screened for eligibility, 170 (46%) were included in the primary analysis (85 pre- and 85 post-intervention). The leading reasons for exclusion were presence of concomitant infection and receipt of antibiotics immediately prior to admission. Urinalyses were ordered most frequently by an emergency medicine provider (80% vs. 69.4%). Urinary symptoms were reported in 27% of pre-intervention patients and 29.4% of post-intervention patients. The primary outcome of antibiotic days per 1000 patient days was 190 days and 159 days, pre- and post-intervention (percent change -16.3%; p=0.568). Empiric and definitive antibiotic coverage rates increased by 46.4% and 36.8% respectively in the post-intervention period.

**Conclusion:**

This initiative was associated with a decrease of 31 antibiotic days per 1000 patient days, a clinically, although not statistically, significant decrease in antimicrobial use and uncovered areas for future intervention focus and research.

**Disclosures:**

Milner Staub, MD, MPH, Eli Lilly: Stocks/Bonds (Public Company)|Gilead: Stocks/Bonds (Public Company)|Johnson & Johnson: Stocks/Bonds (Public Company) Maria N. Muehrcke, PharmD, MBA, BCPS, Pfizer Inc.: Stocks/Bonds (Public Company) Laura Bobbitt, PharmD, Matinas: Stocks/Bonds (Public Company)

